# Senolytic treatment to rescue hallmarks of senescence in lymph node fibroblasts from patients with rheumatoid arthritis: Implications for premature aging and potential therapeutic intervention in early rheumatoid arthritis

**DOI:** 10.1093/cei/uxaf029

**Published:** 2025-05-08

**Authors:** T A de Jong, J F Semmelink, J W Bolt, C Grasso, R A Hoebe, P M Krawczyk, L G M van Baarsen

**Affiliations:** Department of Rheumatology & Clinical Immunology and Laboratory for Experimental Immunology, Amsterdam UMC Location University of Amsterdam, Amsterdam, The Netherlands; Inflammatory Diseases, Amsterdam Institute for Infection and Immunity, Amsterdam, The Netherlands; Amsterdam Rheumatology & Immunology Center (ARC), Academic Medical Center, Amsterdam, The Netherlands; Department of Rheumatology & Clinical Immunology and Laboratory for Experimental Immunology, Amsterdam UMC Location University of Amsterdam, Amsterdam, The Netherlands; Inflammatory Diseases, Amsterdam Institute for Infection and Immunity, Amsterdam, The Netherlands; Amsterdam Rheumatology & Immunology Center (ARC), Academic Medical Center, Amsterdam, The Netherlands; Department of Rheumatology & Clinical Immunology and Laboratory for Experimental Immunology, Amsterdam UMC Location University of Amsterdam, Amsterdam, The Netherlands; Inflammatory Diseases, Amsterdam Institute for Infection and Immunity, Amsterdam, The Netherlands; Amsterdam Rheumatology & Immunology Center (ARC), Academic Medical Center, Amsterdam, The Netherlands; Department of Rheumatology & Clinical Immunology and Laboratory for Experimental Immunology, Amsterdam UMC Location University of Amsterdam, Amsterdam, The Netherlands; Inflammatory Diseases, Amsterdam Institute for Infection and Immunity, Amsterdam, The Netherlands; Amsterdam Rheumatology & Immunology Center (ARC), Academic Medical Center, Amsterdam, The Netherlands; Department of Medical Biology, Amsterdam UMC Location, University of Amsterdam, Cancer Center Amsterdam, Amsterdam, The Netherlands; Department of Medical Biology, Amsterdam UMC Location, University of Amsterdam, Cancer Center Amsterdam, Amsterdam, The Netherlands; Department of Rheumatology & Clinical Immunology and Laboratory for Experimental Immunology, Amsterdam UMC Location University of Amsterdam, Amsterdam, The Netherlands; Inflammatory Diseases, Amsterdam Institute for Infection and Immunity, Amsterdam, The Netherlands; Amsterdam Rheumatology & Immunology Center (ARC), Academic Medical Center, Amsterdam, The Netherlands; Tissue Function and Regeneration, Amsterdam Movement Sciences, Amsterdam, The Netherlands

**Keywords:** autoimmunity, RA-risk individuals, lymph node fibroblasts, senescence

## Abstract

Cellular senescence, a state of proliferation arrest, is implicated in the pathogenesis of age-related diseases such as rheumatoid arthritis (RA). The pathogenesis of RA, characterized by immune dysregulation and systemic autoimmunity preceding clinical onset of disease, may involve early accumulation of senescent lymph node (LN) fibroblasts driving immune tolerance breakdown. This study aims to explore the hallmarks of senescence in LN fibroblasts during the earliest phases of RA and evaluate the effects of dasatinib. Human LN fibroblasts were isolated from inguinal LN needle biopsies from autoantibody-positive individuals at risk of developing RA (RA-risk individuals), RA patients, and seronegative healthy volunteers. Senescence hallmarks and the effects of dasatinib treatment were assessed using quantitative gene expression analysis, flow cytometry, microscopy, and live-cell imaging. Cell size, granularity, and autofluorescence were significantly greater in RA LN fibroblasts compared with controls. Altered gene expression of senescence-associated genes was observed in RA LN fibroblasts. Elevated senescence-associated β-galactosidase activity, more lipofuscin-positive granules, and DNA damage were observed in RA-risk and RA LN fibroblasts. Notably, RA(-risk) LN fibroblasts presented impaired DNA damage repair capacity. Dasatinib treatment significantly improved the size and ability of the LN fibroblast pool to repair DNA damage. We observed multiple senescence hallmarks in RA LN fibroblasts and, to a lesser extent, in RA-risk LN fibroblasts, which could be partially restored by senescent cell removal via dasatinib treatment. These findings suggest a role for senescent LN fibroblasts in RA pathogenesis and highlight the potential of dasatinib as a potential therapeutic intervention to mitigate senescence-associated defects in RA.

## Teaser

This study aims to explore the hallmarks of senescence in lymph nofde (LN) fibroblasts during the earliest phases of rheumatoid arthritis (RA) and evaluate the effects of dasatinib. Human LN fibroblasts were isolated from inguinal LN needle biopsies from autoantibody-positive individuals at risk of developing RA (RA-risk individuals), RA patients, and seronegative healthy volunteers. Senescence hallmarks and the effects of dasatinib treatment were assessed using quantitative gene expression analysis, flow cytometry, microscopy, and live-cell imaging.

## Graphical Abstract

**Graphical Abstract:**
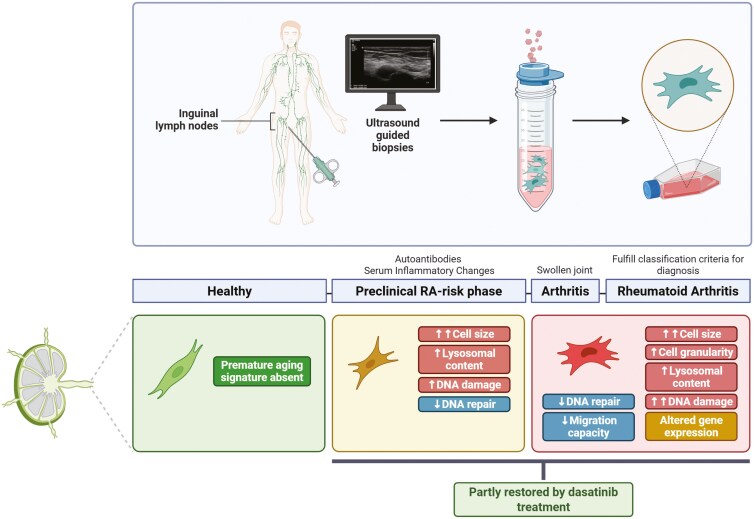

## Introduction

Aging and age-related diseases have become more prevalent since the average human life span has drastically increased. One hallmark of aging is the accumulation of senescent cells [1]. These cells are in a state in which they are viable and metabolically active, yet do not proliferate. Cellular senescence can be described as an emergency brake on cells that allows proper repair during cell cycle arrest to prevent tumorigenesis [2, 3]. However, the persistence and accumulation of senescent cells can cause tissue imbalance and have been implicated in the pathogenesis of age-related diseases such as rheumatoid arthritis (RA) [4, 5].

Aging is an important risk factor for RA, a systemic chronic autoimmune disease in which immune cells infiltrate synovial tissue [6]. Age-associated changes in immune function contribute to cellular senescence and create a pro-inflammatory microenvironment that might accelerate disease progression in RA [5, 7]. Previous studies have characterized premature aging of T cells in RA patients and have shown that maladaptive aging of T cells contributes to the development of autoimmunity [8, 9]. Moreover, it has been shown that synovial fibroblasts from RA patients display pro-inflammatory and senescent characteristics and accumulate in synovial tissues [10]. These studies show that senescence in RA is not limited to one cell type. As most studies have been performed using tissues from patients with established disease, it is unclear whether senescence is a primary cause of RA or secondary to chronic inflammation.

In the absence of synovial inflammation [11, 12], RA-specific autoantibodies such as rheumatoid factor and anti-citrullinated protein antibodies (ACPAs) can be present years before clinical manifestation of disease [13–15]. This allows the identification of individuals at risk of developing RA (RA-risk) [16] and enables studies on the preclinical phase of disease in the absence of inflammation. Because systemic autoimmunity apparently precedes synovial tissue inflammation, it is hypothesized that other unidentified immune processes, possibly outside the synovium, are altered and contribute to disease development. Because autoimmunity can develop when tolerance mechanisms are not properly controlled in secondary lymphoid organs, studying these in the at-risk phase of RA might be important. In secondary lymphoid organs, such as peripheral lymph nodes (LNs), the modulation of effective immune responses and the regulation of peripheral tolerance depend on proper functioning of LN fibroblasts through their production of key chemokines, cytokines, extracellular matrix, as well as the presentation of self-antigens [17–21]. Previously, we showed that LN fibroblasts from patients with RA have a reduced capacity to respond to external triggers such as TLR-3 and TNF [22, 23]. We hypothesize that during the development of RA, LN fibroblasts might exhibit a senescent phenotype and accumulate prematurely in lymphoid organs, which can potentially lead to defective peripheral tolerance, improper control of immune responses, and the development of systemic autoimmune disease.

To date, the etiology of RA remains unclear, and although current treatment options are able to suppress inflammation and joint pain, curative treatment does not exist. To target senescent cells, there are several senolytic agents that selectively eliminate senescent cells by inducing apoptosis. Dasatinib, a tyrosine kinase inhibitor, has been shown to attenuate arthritis symptoms in murine collagen-induced arthritis [24]. Additionally, *in vitro* cultured synovial fibroblasts from RA patients showed decreased proliferation and migration capacity upon dasatinib treatment. Moreover, dasatinib treatment led to a significant increase in apoptosis of synovial fibroblasts [24]. Transcriptional analysis comparing peripheral blood-derived T cells from RA patients and age-matched healthy controls revealed that ephrin B1 (*EFNB1*), a tyrosine kinase ligand targeted by dasatinib, was elevated in RA patients. Furthermore, *EFNB1* expression correlated with RA symptoms [25], making dasatinib an interesting drug for potential future therapies. In this study, we investigated the senescence phenotype of LN fibroblasts during health, systemic autoimmunity, and RA. Additionally, we examined whether dasatinib treatment could restore the cellular phenotype and function of the pool of LN fibroblasts.

## Materials and methods

### Study subjects and tissue samples

Individuals with arthralgia and/or a family history of RA who were positive for ACPAs (detected by the anti-cyclic citrullinated peptide [anti-CCP] antibody test [CCPlus anti-cyclic citrullinated peptide 2 ELISA {ULN 25 kAU/L}] Eurodiagnostica, Nijmegen, The Netherlands) and without any evidence of arthritis upon examination were included. These individuals are considered to be at risk of developing RA (RA-risk individuals), characterized by the presence of systemic autoimmunity associated with RA but without clinical arthritis (defined as phase c + d, according to EULAR recommendations) [16, 26]. After a median follow-up of 20 months (11–46 (interquartile range [IQR]), none of these individuals developed RA despite the presence of ACPAs. However, we expect that 30% of these individuals will eventually develop arthritis within 3–4 years [13–15]. For comparison, we included patients diagnosed with RA (ACR/EULAR 2010 criteria) [26] who were all biological naïve and ACPA positive, and while two patients had longstanding RA (>8 years), three had a recent diagnosis. Finally, age-matched seronegative healthy volunteers without any history of autoimmunity or inflammatory disease and no present or previous use of disease-modifying antirheumatic drugs (DMARDs) or biologicals were included. Study subjects were recruited either via the outpatient clinic of the Department of Rheumatology and Clinical Immunology at the Amsterdam UMC via referral from the rheumatology outpatient clinic of Reade, Amsterdam, or by testing family members of RA patients in the outpatient clinic. LN tissues were collected via ultrasound-guided inguinal LN needle core biopsy as previously described [27]. The study was performed according to the principles of the Declaration of Helsinki [28] and approved by the Institutional Review Board of the Amsterdam UMC, and all study subjects provided written informed consent. Table 1 shows the demographics of the included study subjects.

**Table 1:** demographic baseline characteristics of study subjects

	*Ex vivo* cohort			Cell culture cohort		
	Controls	RA-risk individual	RA patients	Controls	RA-risk individual	RA patients
	*n* = 9	*n* = 4	*n* = 11	*n* = 5	*n* = 5	*n* = 5
Sex (female, *n*) (%)	5 (56)	2 (50)	5 (45)	3 (60)	4 (80)	4 (80)
Age (years)	47 (37–63)	51 (41–57)	69 (59–72)	47 (33–58)	48 (36–50)	42 (29–44)
IgM-RF positive (*n*) (%)	ND	3 (75)	7 (64)	0 (0)	0 (0)	4 (80)
ACPA positive (*n*) (%)	ND	3 (75)	9 (82)	0 (0)	5 (100)	5 (100)
ESR (mm/h)	ND	6.5 (2.8–8)	12 (5–22)	ND	5 (2–8.5)	26 (6.5–40.5)
CRP	ND	0.7 (0.5–1.9)	3.8 (1.1–8)	0.5 (0.3–3.9)	1.6 (0.8–3.6)	3 (1.7–26.5)

Abbreviations: ACPA, anti-citrullinated protein antibodies; CRP, C-reactive protein; ESR, erythrocyte sedimentation rate; IgM-RF, IgM rheumatoid factor; ND, not determined; VAS, visual analogue scale.

Data are expressed as median (interquartile range) unless otherwise indicated. IgM-RF: IgM rheumatoid factor (measured via IgM-RF ELISA, ULN 49 IU/ml; Hycor Biomedical, Indianapolis, IN).

### Flow cytometry for direct *ex vivo* fibroblast analyses

For direct *ex vivo* comparison, LNs from kidney transplantation recipients were collected and carefully cleaned of fat and connective tissue. Subsequently, LNs were cut into small pieces (<0.5 cm^2^) after which LN tissue samples (from kidney transplantation and inguinal core needle biopsies) were enzymatically digested three times for 15 minutes each. The digestion mixture consisted of 0.2 mg/ml collagenase P, 0.8 mg/ml dispase II, and 0.1 mg/ml DNase I (all Roche, Woerden, The Netherlands) in RPMI (Invitrogen, Landsmeer, The Netherlands). After every digestion step, the cell suspension was filtered through a 100-m nylon cell strainer (BD Falcon, San Jose, CA), and after the final step, the cells were washed and collected in PBA buffer (PBS containing 0.5% BSA and 0.01% NaN_3_ [Sigma Aldrich, Zwijndrecht, The Netherlands]). Next, cells were stained for 60 minutes at 4°C with unconjugated anti-PDPN (NC-08, Angiobio). After incubation, cells were washed twice in PBA buffer and stained for 30 minutes with fluorochrome-labeled antibodies against the following markers: CD31-Alexa Fluor 488 (clone WM-59, Biolegend, San Diego, CA), HLA-DR-PE (clone L243, eBioscience), Alexa Fluor 647 (polyclonal, Invitrogen), CD235a-eFluor450 (clone HIR2, eBioscience), CD45-eFluor450 (clone HI30, eBioscience), PD-L1-BV510 (clone 29E.2A3), CD86-BV650 (clone IT2.2, Biolegend), CD80-PEcy7 (clone 2D10, Biolegend), CD40-PEdazzle594 (clone 5C3, Biolegend), CD11c-AlexaFluor700 (clone Bu15, Biolegend), and viability dye-eFluor780 (Invitrogen). Subsequently, cells were washed twice in PBA buffer and analyzed using the BD FACS Aria SORP Cell Sorter. Flow cytometry data were analyzed using FlowJo 10.8.1 (Tree Star, Ashland, OR).

### Fibroblast culture, senescence induction, and dasatinib treatment

LN fibroblasts were isolated and expanded *in vitro* as previously described, resulting in cultures containing LN fibroblasts [23]. Experiments were performed using cultured human LN fibroblasts between passages 5 and 7. To induce DNA damage, control LN fibroblasts were irradiated in culture flasks at 10 Gy via CellRad + (Precision X-ray, Madison, CT) and passaged simultaneously with nonirradiated cells from the same donor. RA-risk, RA, and irradiated control LN fibroblasts were treated with 5 µM dasatinib (MedChemExpress, Monmouth Junction, NJ) for 24 hours to eliminate senescent cells. After 24 hours of treatment, culture flasks were washed with complete cell culture media and cultured to 80% confluence, based on the flask containing untreated cells, after which all cells were harvested simultaneously for analyses.

For analyses, cultured LN fibroblasts were non-enzymatically detached with TrypLE Select (Gibco, Bleiswijk, The Netherlands) for 7 minutes at 37°C and collected for flow cytometry experiments. Cells were washed and collected in PBA buffer (PBS containing 0.5% BSA and 0.01% NaN_3_ [Sigma Aldrich, Zwijndrecht, The Netherlands]). Subsequently, LN fibroblasts were measured on a Spectral Analyzer SP6800 (Sony Biotechnology, Weybridge, United Kingdom) and analyzed using FlowJo 10.8.1 (TreeStar Inc., Ashland, OR). Cell size and granularity were determined based on the parameters FSC-W and SSC-A, respectively.

### Quantitative real-time PCR

Total RNA was isolated using the RNA micro kit (Qiagen, Venlo, The Netherlands) according to the manufacturer’s instructions. Subsequently, cDNA was prepared using the RevertAid H Minus First Strand cDNA Synthesis Kit (Thermo Fisher Scientific, Landsmeer, The Netherlands). Quantitative PCR was performed using either Taqman Gene Expression master mix combined with Taqman assays or fast SYBR Green PCR master mix (all Applied Biosystems, Life Technologies, Zwijndrecht, The Netherlands) combined with in-house-designed primers (Thermo Fisher). Primer sequences and Taqman assays are described in Table 2. For detection, we used QuantStudio 3 (Applied Biosystems). Values of each target gene were normalized to the geometric mean expression levels of two reference genes: *RPLP0* and *POLR2G*. An arbitrary calibrator sample was used to correct for inter-plate differences. To calculate the relative quantity, the standard curve method was applied to SYBR Green assays, whereas the delta‒delta Ct method was applied to Taqman assays.

**Table 2.** overview of primer sequences

SYBRgreen			
Gene	mRNA transcriptID	Forward sequence	Reverse sequence
*POLR2G*	NM_002696.3	GAGGTCGTGGATGCTGTTGT	TCTCTGAAGGGATGGAATGTCG
*RPLP0*	NM_001002.4	GCAGCATCTACAACCCTGAAGT	GCAGACAGACACTGGCAACAT
*FOXO4*	NM_001170931.1	GGAAAAGGCCATTGAAAGCG	ATGAACTTGCTGTGCAGGGA
*TP53*	NM_001126116.1	CAGTCACAGCACATGACGGA	GCCAGACCATCGCTATCTGAG
*CDKN1A*	NM_078467.2	AGACCAGCATGACAGATTTCTACC	GCGGATTAGGGCTTCCTCTT
*CDKN2A*	NM_000077.4	TCCCTCAGACATCCCCGATT	CCTGTAGGACCTTCGGTGAC
*GLB1*	NM_001079811.2	TTTGCTCTGCGAAACATCATCC	GCTCCCACTGTCTTTAACTTTTCC
*BCL2L1*	NM_138578.3	CTGTGCGTGGAAAGCGTAGA	GCTGCTGCATTGTTCCCATAG
*SIRT1*	NM_001314049.1	GAGCAGATTAGTAGGCGGCTT	CTCAGCGCCATGGAAAATGT
*NAMPT*	NM_005746.3	TCTGGAAACCCTCTTGACACTG	GTTTCATGCCTTCTACAATCTCTTG
*PARP1*	NM_001618.4	AACCGAAGATTGCTGTGGCA	ACCAAACATGTAGCCTGTCACG
*CD38*	NM_001775.4	GGTGGAAGAGAAGATTCCAGAGAC	TAAAACAACCACAGCGACTGG
*EFNB1*	NM_004429.5	GGAGGCAGACAACACTGTCA	TCCTGGTTCACAGTCTCATGC
*LMNB1*	NM_001198557.1	AAATTCTCAGGGAGAGGAGGT	TTGGATGCTCTTGGGGTTC
**Taqman assays**			
**Gene**	**Assay ID**		
*POLR2G*	Hs00275738_m1		
*RPLP0*	Hs00420895_gH		
*NOTCH3*	Hs01128537_m1		

### Senescence, DNA damage, and repair analyses

LN fibroblasts were seeded in triplicate at a density of 25 000 cells/well on sterilized round 12-mm coverslips (Knittel-Glaeser, Bielefeld, Germany) in 24-well plates (Corning, Amsterdam, The Netherlands) and cultured for 24 hours in complete culture medium. After 24 hours, cells were washed with PBS and fixed with 4% paraformaldehyde (PFA) for 10 minutes. After washing with PBS, cells were permeabilized for 60 minutes at room temperature (RT) with PBS containing 1% BSA and 0.1% saponin (Sigma‒Aldrich) and stained overnight at 4°C with monoclonal mouse IgG anti-human yH2AX (Ser139, Sigma‒Aldrich). The next day, cells were washed with PBS + 1% BSA + 0.1% saponin and incubated for 30 minutes at 4°C with secondary anti-mouse IgG1 AlexaFluor633 (Invitrogen, Landsmeer, the Netherlands). Subsequently, coverslips were removed from the wells and left to dry at RT in the dark. Coverslips were mounted on microscope slides with DAPI Vectashield Hardset mounting media (Vector Laboratories). yH2AX staining, which represents DNA damage at baseline, was analyzed using confocal microscopy (TCS SP8, Leica Microsystems). DNA damage repair analysis was subsequently performed on irradiated cells (1 Gy), followed by yH2AX staining either directly or 3 or 24 hours after irradiation. DNA damage repair was visualized using a Leica DMi8 microscope, and staining intensity was analyzed in approximately 50 cells/donor using LAS X 3D and QuPath (v0.3.2).

The lysosomal content was measured by staining for lipofuscin granules and senescence-associated β-galactosidase (SA-β-gal) activity in cultured LN fibroblasts. Lipofuscin granules were stained with SenTraGor reagent (Lab Supplies Scientific, Athens, Greece). A total of 25 000 cells/well (for lipofuscin) or 10 000 cells/well (for SA-β-gal) were seeded in triplicate on round 12-mm coverslips in 24-well plates and cultured in a 37°C/5% CO_2_ incubator for 24 hours. After 24 hours, immunofluorescence staining with SenTraGor was performed according to the manufacturer’s instructions [29]. SA-β-gal was detected via a senescence detection kit (Abcam, Cambridge, UK) according to the manufacturer’s instructions. Subsequently, coverslips were removed from the wells and mounted on microscope slides with DAPI Vectashield Hardset mounting media. For analysis of lipofuscin granules and SA-β-gal, approximately 50 cells/donor were imaged using confocal microscopy (TCS SP8, Leica Microsystems, Amsterdam, The Netherlands) and wide-field microscopy (DM6, Leica) and analyzed using Las X 3D and the Senescence Counter macro in ImageJ [30].

### Cellular viability and live-cell analysis of proliferation and migration

Cellular viability was measured via the MTT reagent (3-[4,5-dimethylthiazol-2-yl]-2,5-diphenyltetrazolium bromide; thiazolyl blue, Sigma). LN fibroblasts were seeded at 2500 cells/well in 96-well plates, and an MTT assay was performed 24, 72, and 120 hours after seeding. MTT stock solution (5 mg/ml) was added to a 1:10 dilution of the original culture medium, and the mixture was incubated for 2 hours. After incubation, the culture medium was removed, and the converted dye was solubilized with acidic isopropanol and 4 mM HCl + 0.1% NP-40 (Calbiochem, San Diego, CA) in absolute isopropanol. The absorbance of the converted dye was measured via a spectrophotometer (VersaMax, Molecular Devices, San Jose, CA) at 580 nm.

Cellular proliferation and migration of cultured LN fibroblasts were monitored over time via an IncuCyte S3 ZOOM live-cell imaging microscope. For proliferation, cells were seeded at a density of 2500 cells/well in 96-well plates and incubated in the IncuCyte for 6 days. For migration, LN fibroblasts were seeded at 10 000 cells/well in a 96-well ImageLock plate (Sartorius, Goettingen, Germany) and cultured overnight in a 37°C/5% CO_2_ incubator. The next day, a scratch wound was created using the IncuCyte wound maker according to the manufacturer’s instructions. After wounding, cells were washed with PBS, and fresh complete cell culture media was added. Culture plates were incubated in the IncuCyte for 5 days. Relative wound density, which reflects the ratio of the occupied area to the total area of the initial scratched region, and confluence were analyzed using the Incucyte software.

### Statistics

Data are presented as median with interquartile range (IQR). Statistical differences between the study groups at baseline were analyzed using a Kruskal–Wallis test followed by a post hoc Dunn’s test, a two-way ANOVA or repeated-measures ANOVA test with Geisser–Greenhouse correction followed by a Dunnett’s multiple comparison test, where appropriate. A Wilcoxon matched pairs signed-rank test was used to analyze the effects of irradiation and dasatinib treatment. GraphPad Prism software (version 9.1.0; La Jolla, CA, USA) was used for statistical analysis. *P*-values <0.05 were considered statistically significant.

## Results

### LN fibroblasts from RA patients have increased size and granularity

**Figure 1:**
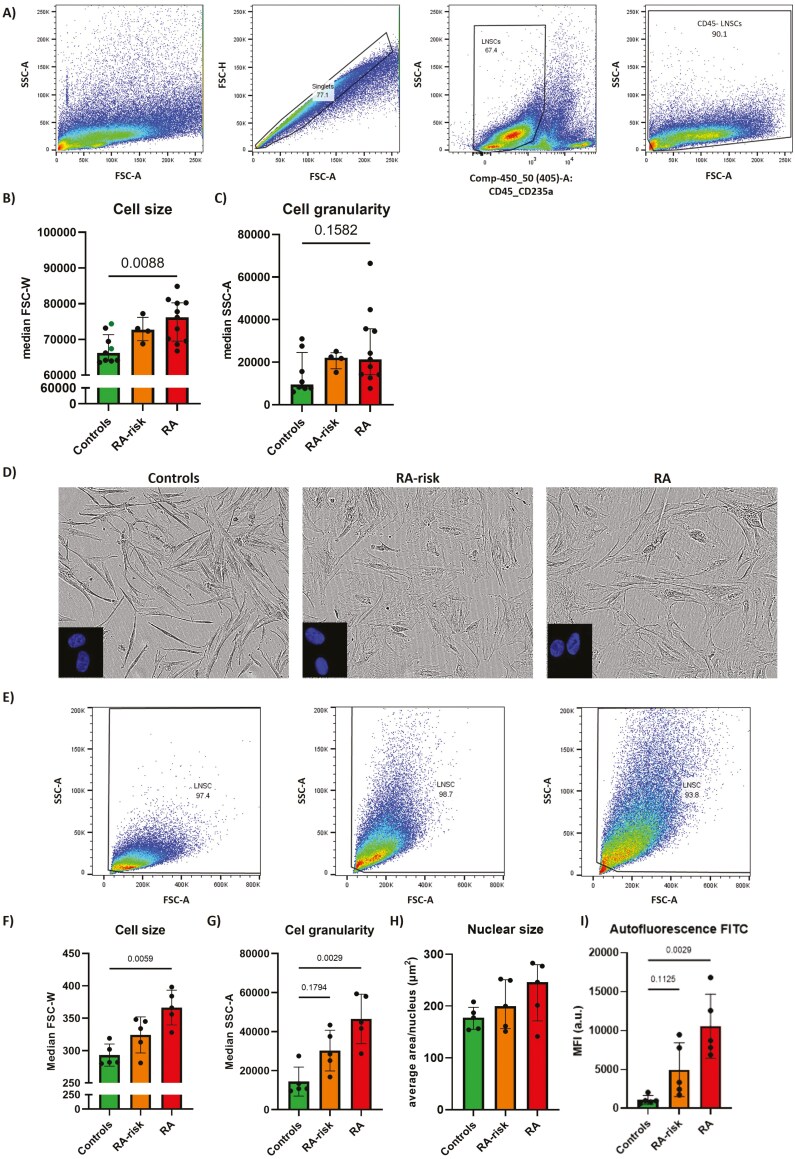
morphological alterations in directly ex vivo analyzed and cultured RA LN fibroblasts compared with controls. (A) Flow cytometry gating strategy used to identify single cells (FSC-H vs. FSC-A), CD45^-^CD235^-^ cells (SSC-A v.s CD45/CD235a), and CD45^-^ LN fibroblasts. Numbers adjacent to the outlined areas indicate the percentages of cells in the gated population. (B) Cell size and (C) cell granularity measured by flow cytometry. Green = non-kidney transplant healthy volunteers undergoing LN needle biopsy. (D) Representative phase-contrast images of cultured LN fibroblasts generated via IncuCyte ZOOM. Insert: representative immunofluorescent stainings of LN fibroblasts nuclei using DAPI. (E) Representative flow cytometry plots showing the LN fibroblast population on forward–sideward scatter. (F) Cell size measured by flow cytometry (MFI of FSC-W). (G) Cell granularity (MFI of SSC-A). (H) Nuclear size of cultured LN fibroblasts measured by immunofluorescent DAPI staining; approximately 50 cells per donor were measured for analysis via LAS X 3D and ImageJ. (I) Autofluorescence of unstained cells in the FITC channel. All donors were at passage 5 for flow cytometry and at passage 6 for nuclear size measurements. *N* = 5 per group. Data are presented as median + interquartile range. Statistical differences were determined using a Kruskal–Wallis test followed by Dunn’s multiple comparisons test.

Senescent cells exhibit several morphological alterations and are described as having an enlarged and irregular cell shape [31]. Flow cytometry gating strategy used to identify LN fibroblasts is shown in Fig. 1A. Quantitative analysis of LN fibroblasts *ex vivo* revealed that RA LN fibroblasts are significantly larger compared with control LN fibroblasts (Fig. 1B). Cell size of LN fibroblasts from RA-risk individuals was numerically, but nonsignificantly, higher compared with control LN fibroblasts. Cell granularity was not significantly different between diagnoses (Fig. 1C). This suggests that morphological alterations can be observed in LN fibroblasts directly *ex vivo*.

Additionally, *in vitro* cultured LN fibroblasts from RA patients and RA-risk individuals also presented morphological differences compared with control LN fibroblasts on both microscopy images and spectral flow cytometry plots (Fig. 1D and E). Flow cytometry measurements of unstained LN fibroblasts revealed that RA LN fibroblasts harbor a significantly larger cell size and greater granularity than control LN fibroblasts (Fig. 1F and G). Immunofluorescence staining did not reveal any significant differences in nuclear size between the donor groups (Fig. 1H). Increased autofluorescence was recently discussed as a reliable senescence marker for *in vitro* cultured human mesenchymal stromal cells, as it correlates with multiple established hallmarks of senescence [32]. In our cohort, autofluorescence in the FITC channel was significantly higher in RA LN fibroblasts compared with control LN fibroblasts (Fig. 1I). Autofluorescence was detected at similar levels in other fluorescent channels (data not shown). Taken together, these data revealed that the *ex vivo* increase in cell size is maintained during LN fibroblast culture, allowing functional *in vitro* studies to assess additional senescence hallmarks.

### Increased lysosomal content in RA(-risk) LN fibroblasts

**Figure 2:**
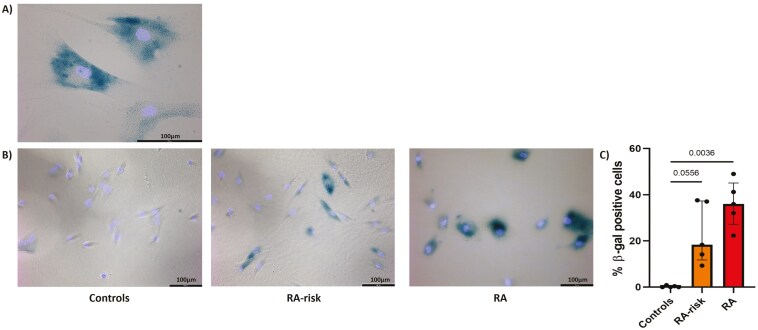
significantly higher SA-β-gal activity in RA-risk and RA LN fibroblasts compared with controls. (A) Representative image reflecting positive and negative SA-β-gal staining when the Senescence Counter macro in ImageJ was used. (B) Representative images of SA-β-gal (dark blue) combined with DAPI nuclear staining (light blue in cultured LN fibroblasts. C) SA-β-gal-positive LN fibroblasts as a percentage of DAPI-positive cells. All donors at passage 6, *n* = 5 per group and approximately 50 cells per donor, were analyzed. Data are presented as median + interquartile range. Statistical differences were determined using a Kruskal–Wallis test followed by Dunn’s multiple comparisons test.

We next evaluated the lysosomal content of cultured LN fibroblasts by measuring the activity of senescence-associated β-galactosidase (SA-β-gal), a lysosomal enzyme. Cultured LN fibroblasts were defined as SA-β-gal positive at a cutoff intensity according to the Senescence Counter macro in ImageJ, as shown in Fig. 2A. Hardly any positive cells were detected in control LN fibroblasts, whereas RA LN fibroblasts presented a relatively high percentage of blue-stained cells (Fig. 2B). Image quantification revealed that SA-β-gal activity was significantly higher in RA LN fibroblasts and numerically higher in RA-risk LN fibroblasts compared with controls (Fig. 2C).

### Morphological alterations are partially restored by dasatinib treatment

Because we detected relatively high SA-β-gal positivity in cultured LN fibroblasts, we investigated whether LN fibroblasts are sensitive to senolytics that specifically target senescent cells. The most studied senolytic agent in fibroblasts is Navitoclax, which induces apoptosis by targeting the antiapoptotic pathways of BCL2 and BCL-xL. However, titration experiments in LN fibroblasts did not show any effect on cell viability (Supplementary Fig. 1A), suggesting that LN fibroblasts are not sensitive to Navitoclax. Similarly, the mRNA levels of *BCL2L1* (encoding Bcl-xL) were not differentially expressed between donor groups (Supplementary Fig. 1B). The senolytic piperlongumine decreased cell viability in both control and RA LN fibroblasts (Supplementary Fig. 1C), making it an unsuitable drug for targeting altered LN fibroblasts in RA patients, and quercetin did not have any effect (Supplementary Fig. 1D). In contrast, dasatinib, which has been reported to effectively eliminate senescent preadipocytes [33], reduced cell viability only in RA LN fibroblasts (Supplementary Fig. 1E). Dasatinib is often used in combination with quercetin, but we did not detect an additional decrease in cell viability after combining the two senolytic drugs (data not shown). Dasatinib interferes with ephrin B1 (EFNB1), an ephrin-dependent receptor ligand, which was nonsignificantly higher expressed in RA LN fibroblasts compared with control LN fibroblasts (Supplementary Fig. 1F). Guided by these findings, showing a selective effect of dasatinib treatment on RA LN fibroblasts, we next investigated the effect of dasatinib treatment on cell morphology.

**Figure 3:**
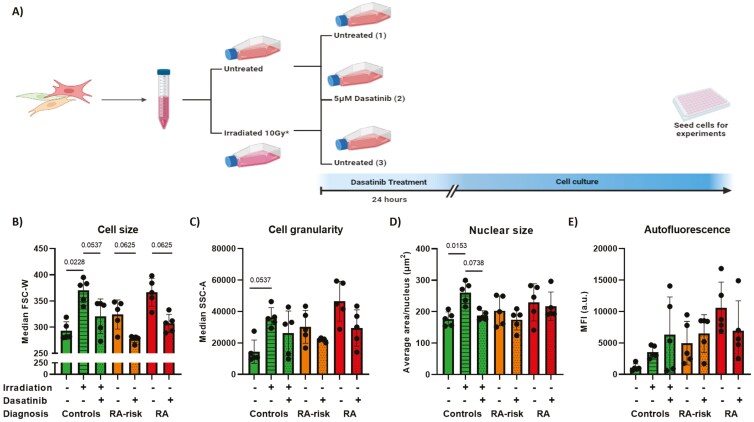
dasatinib treatment partially restored cell size in RA-risk and RA LN fibroblasts. (A) Graphical overview of dasatinib treatment experimental setup. After cell collection, RA-risk and RA LN fibroblasts were seeded in two flasks, and controls were seeded in three flasks. The next day, one flask was treated with 5 µM dasatinib for 24 hours, and the other was left untreated. After 24 hours, dasatinib was removed, and the remaining cells were left in culture until the flasks with untreated LN fibroblasts reached 80% confluence. Upon reaching confluence, both flasks per donor were harvested and seeded for experiments. Two flasks of control LN fibroblasts were irradiated with 10 Gray, one of which was treated for 24 hours with 5 µM dasatinib 24 hours after irradiation, and the other was left only irradiated. The third control LN fibroblast flask was untreated, and once these LN fibroblasts were confluent, the other two flasks were harvested and plated for experiments. *Only control LN fibroblasts. (B) Cell size, (C) cell granularity, (D) nuclear size (approximately 50 cells per donor were measured for analysis), and (E) autofluorescence of RA-risk and RA LN fibroblasts before and after dasatinib treatment and control LN fibroblasts before and after irradiation and dasatinib treatment. All donors were at passage 5 for flow cytometry and at passage 6 for nuclear size measurements. Data are presented as median + interquartile range, and a Wilcoxon matched pairs signed-rank test was used to analyze the effect of dasatinib treatment.

To selectively eliminate senescent cells, cultured RA-risk and RA LN fibroblasts were treated with 5 µM dasatinib for 24 hours. Subsequently, dasatinib was removed, after which the remaining cells were allowed to proliferate until the untreated cells reached confluence, after which the cells were harvested and analyzed (Fig. 3A). To demonstrate the functional effect of dasatinib on senescent cells, healthy LN fibroblasts were irradiated before treatment, which, as expected, led to a significant increase in cell and nuclear size and a numerical increase in cell granularity and autofluorescence (Fig. 3B–E and Supplementary Fig. 2). Removal of senescent cells by dasatinib treatment partly improved the morphological alterations observed in RA-risk and RA LN fibroblasts, as well as in irradiated healthy LN fibroblasts (Fig. 3B–E).

### Dasatinib eliminates LN fibroblasts containing increased lysosomal biogenesis

**Figure 4:**
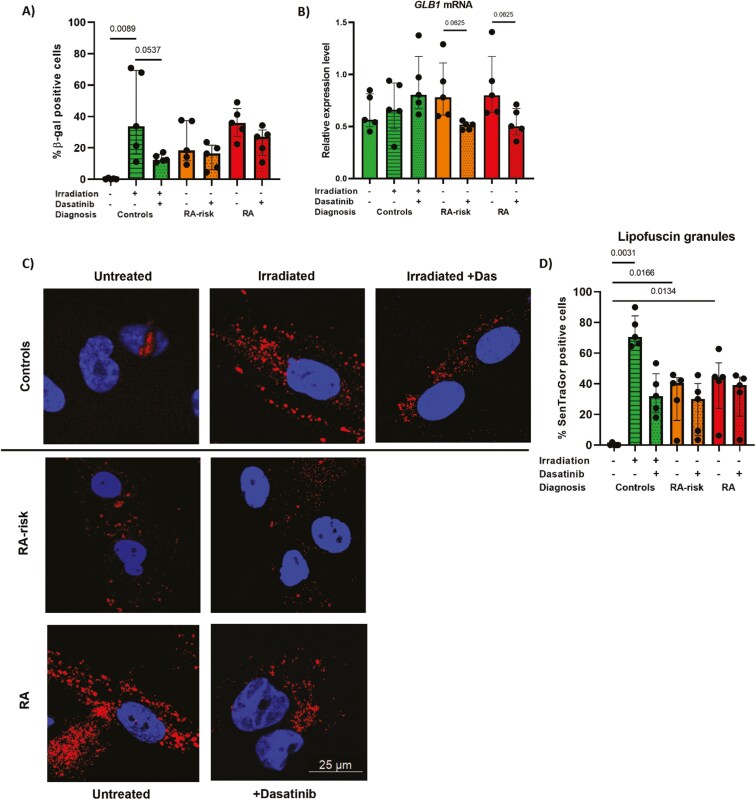
dasatinib treatment restores increased lysosomal accumulation in LN fibroblasts. (A) SA-β-gal-positive LN fibroblasts as a percentage of DAPI-positive cells before and after treatment with 5 µM dasatinib. (B) Relative gene expression level of *GLB1.* (C) Representative images of SenTraGor-stained lipofuscin granules (red) and DAPI-stained nuclei (blue) in cultured LN fibroblasts. (D) Percentage of SenTraGor-positive cells relative to DAPI-positive cells as measured by LAS X 3D and ImageJ. All donors at passage 6, *N* = 5 per group, approximately 50 cells per condition, were analyzed. Data are presented as median + interquartile range. Statistical differences at baseline were determined using a Kruskal–Wallis test, followed by Dunn’s multiple comparisons test and a Wilcoxon matched pairs signed-rank test was used to analyze the effect of dasatinib treatment.

We next evaluated whether the observed increase in SA-β-gal activity in RA(-risk) LN fibroblasts would also decrease after dasatinib treatment. A significant increase in SA-β-gal-positive cells in irradiated control LN fibroblasts confirmed that those cells acquired a senescent phenotype (Fig. 4A). Dasatinib treatment after irradiation significantly reduced the percentage of SA-β-gal-positive cells in these control LN fibroblasts. Although dasatinib treatment did not significantly affect SA-β-gal activity in either RA- or RA-risk LN fibroblasts, there was a pronounced decrease in the expression level of *GLB1*, the gene encoding SA-β-gal (Fig. 4B).

Another marker for lysosomal biogenesis is SenTraGor, which is used to detect lipofuscin granules. Lipofuscin granule accumulation indicates reduced lysosomal degradation. SenTraGor can be visualized via immunofluorescence and was rarely detected in control LN fibroblasts (Fig. 4C). In line with the SA-β-gal data, irradiation of control LN fibroblasts significantly increased the number of lipofuscin granules, which was reduced after dasatinib treatment (Fig. 4D), suggesting that dasatinib treatment can target lysosomes. The percentage of SenTraGor-positive cells was significantly higher in both RA-risk and RA LN fibroblasts compared with control LN fibroblasts. The ability of dasatinib to target endogenous lipofuscin granules was highly variable within donor groups (Fig. 4D).

### Senescence-associated gene expression profile in RA LN fibroblasts

**Figure 5:**
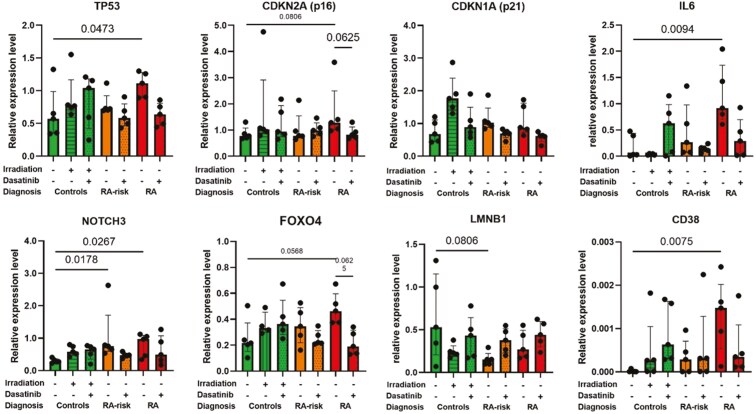
altered gene expression profiles in RA(-risk) LN fibroblasts. Gene expression levels of senescence-associated genes in cultured LN fibroblasts. All donors were at passage 6, *N* = 5 per group. Data are presented as median + interquartile range. Statistical differences at baseline were determined using a Kruskal–Wallis test, followed by Dunn’s multiple comparisons test and a Wilcoxon matched pairs signed-rank test was used to analyze the effect of dasatinib treatment.

Senescent cells accumulate primarily in chronically inflamed tissues, which can have detrimental consequences for tissue structure and regeneration [34, 35]. Senescent cells can create a pro-inflammatory microenvironment by secreting a variety of different molecules, such as interleukins, chemokines, growth factors, and growth regulators, to communicate with adjacent cells. Released factors can affect cell proliferation, disrupt tissue structure and function, and immunomodulation [36, 37]. Indeed, gene expression levels of *TP53*, *CDKN1A*, *IL6*, *FOXO4*, and *CD38* were significantly higher in RA LN fibroblasts compared with control LN fibroblasts (Fig. 5). CD38 is related to coenzyme NAD, a critical metabolic for many cellular processes. However, other genes linked to NAD, *SIRT1, PARP1*, and *NAMPT* were not differentially expressed between donor groups (Supplementary Fig. 3). Expression of *NOTCH3* was already significantly altered in RA-risk LN fibroblasts (Fig. 5). Dasatinib treatment had no significant effect on gene expression levels in cultured LN fibroblasts, although dasatinib restored the expression levels of *TP53*, *CDKN2A*, *CDKN1A*, and *FOXO4* in RA(-risk) LN fibroblasts to the expression levels measured in control LN fibroblasts and partially restored the expression levels of *IL6*, *NOTCH3*, *LMNB1*, and *CD38*. This also shows that, at transcriptional level, senescence can be detected in cultured LN fibroblasts from RA patients, which improved after the removal of senescent cells by dasatinib treatment for most of the analyzed genes.

### Lower migration capacity of RA LN fibroblasts

**Figure 6:**
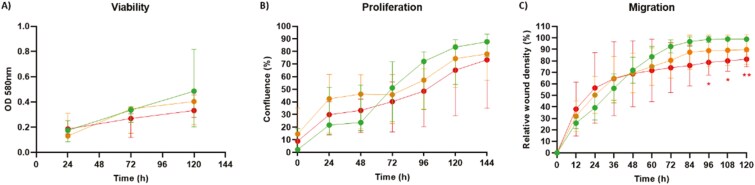
lower migration capacity in RA LN fibroblasts compared with controls. (A) Viability of cultured LN fibroblasts measured via MTT over time. (B) Real-time proliferation of cultured LN fibroblasts measured as confluence via IncuCyte software. (C) Real-time cellular migration capacity of cultured LN fibroblasts measured as relative wound density via the scratch wound assay software from IncuCyte. All donors passage 6, *N* = 5 per group, with three technical replicates per condition for viability and five technical replicates for proliferation and migration assays. Data are presented as median + interquartile range. Statistical differences were determined using a repeated-measures ANOVA with the Geisser–Greenhouse correction followed by Dunnett’s multiple comparisons test. Statistical significance is indicated as *P < 0.05 and **P < 0.01.

Next, we investigated the viability, proliferation, and migration of cultured LN fibroblasts over time. Previously, we showed that, during the first 27 hours after seeding, LN fibroblasts are only attaching and spreading, whereas the proliferation phase starts after these 27 hours [38]. As the viability of the cells was similar between the control and RA(-risk) LN fibroblasts 24 hours after seeding (Fig. 6A), we concluded that, as expected, a similar number of LN fibroblasts were seeded from all donors tested. Over time, the viability of all groups increased, indicating that cell proliferation occurred.

To gain more insight into the proliferation rate of LN fibroblasts in culture, we followed the cells in real time using an IncuCyte ZOOM S3. This system automatically acquired images every 24 hours during a 6-day cell culture experiment and analyzed cell confluency as a measure of the proliferation rate (Fig. 6B). This life-cell analysis confirmed the slow proliferation of LN fibroblasts, which started in control LN fibroblasts 48 hours after seeding. Overall, there was no statistically significant difference in the proliferation rate, but the proliferation rate of RA LN fibroblasts tended to lag behind that of control LN fibroblasts. As a measure of cell function, we also studied the migration capacity of cultured LN fibroblasts via a scratch wound assay. Cells were seeded at confluence, and after 24 hours, an equal scratch wound was made automatically in every well via the IncuCyte wound maker. To analyze the migration capacity, the relative wound density was measured every 12 hours for 5 days, which represented the closing of the wound. Control LN fibroblasts were able to close the wound after 3 days of culture, whereas RA LN fibroblasts never closed the wound, reflecting a significantly lower migration (and/or proliferation) capacity (Fig. 6C).

We next investigated whether dasatinib could impact the observed alterations in cell behavior. As expected, irradiation of control LN fibroblasts had a negative effect on their viability, migration, and proliferation, and dasatinib treatment restored some of these defects (Supplementary Fig. 4). However, dasatinib treatment of RA(-risk) LN fibroblasts did not impact their viability, proliferation, or migration (Supplementary Fig. 4). In summary, these live-cell imaging-based analyses revealed that RA LN fibroblasts have a lower migration and proliferation capacity compared with LN fibroblasts, which could not be restored by the removal of senescent cells via dasatinib treatment.

### RA-risk and RA LN fibroblasts have an impaired capacity to repair DNA damage

**Figure 7.**
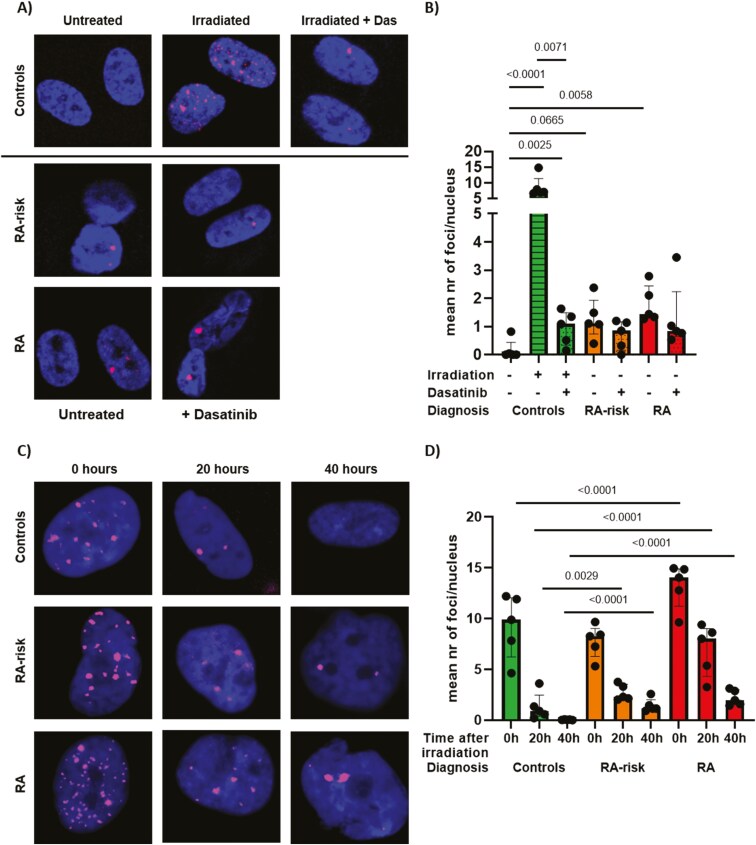
Impaired DNA damage repair in RA(-risk) LN fibroblasts. (A) Representative images of yH2AX foci (pink) and DAPI staining (blue) in cultured LN fibroblasts. (B) Average number of yH2AX foci per nucleus in cultured LN fibroblasts before and after 5 µM dasatinib treatment. All donors were at passage 6 under unstimulated conditions. (C) Representative images of yH2AX foci (pink) and DAPI staining (blue) in cultured LN fibroblasts after DNA damage induction by gamma-irradiation. (B) Average number of yH2AX foci per nucleus in cultured LN fibroblasts directly and 20 and 40 hours after irradiation. Mean value per donor was determined through the quantification of Z-stack images of approximately 50 cells per donor. All donors were at passage 7, *N* = 5 per group. Data are presented as median + interquartile range. Statistical differences were determined using two-way ANOVA + Dunnett’s T3 multiple comparisons test, and a Wilcoxon matched pairs signed-rank test was used to analyze the effect of dasatinib treatment.

We next quantified the number of yH2AX foci that accumulate at sites of unresolved DNA damage. A significantly greater number of foci were observed in the nuclei of RA LN fibroblasts than in those of controls, as was a nonsignificant (*P* = 0.0665) increase in RA-risk LN fibroblasts (Fig. 7A and B). As a positive control, we irradiated LN fibroblasts from controls, which significantly increased the number of yH2AX foci. Treating these irradiated control LN fibroblasts for 24 hours with 5 µM dasatinib significantly reduced the number of yH2AX foci, although not all DNA damage was repaired. These findings indicate that dasatinib treatment can partially eliminate irradiation-induced DNA-damaged LN fibroblasts. However, dasatinib treatment did not decrease the degree of DNA damage present in RA-risk or RA LN fibroblasts (Fig. 7B). Because we already detected DNA damage in cultured LN fibroblasts at baseline, we next investigated whether RA(-risk) LN fibroblasts had impaired DNA repair capacity. Therefore, cultured LN fibroblasts were first irradiated with 1 Gray, after which DNA damage repair was followed over time by imaging these cells directly (0 hour) and 20 and 40 hours after irradiation. Compared with controls, irradiated RA LN fibroblasts presented significantly more DNA damage (Fig. 7C and D), indicating that RA LN fibroblasts might be more sensitive to DNA damage induction. After 20 hours, DNA damage was significantly reduced in all donors, although yH2AX foci levels remained significantly higher in RA-risk and RA LN fibroblasts compared with control LN fibroblasts (Fig. 7C and D), indicating that DNA repair is faster in control LN fibroblasts. Complete DNA damage repair was observed in control LN fibroblasts 40 hours after irradiation, whereas yH2AX foci persisted in RA-risk and RA LN fibroblasts. The number of yH2AX foci detected 40 hours after irradiation in RA-risk and RA LN fibroblasts was still higher than that detected in untreated LN fibroblasts, indicating that DNA damage repair in these cells is slow and incomplete. Pretreatment of cultured LN fibroblasts with 5 µM dasatinib resulted in significantly fewer yH2AX foci directly after irradiation, suggesting that this makes RA-risk and RA LN fibroblasts less sensitive to irradiation-induced DNA damage (Supplementary Fig. 5). Furthermore, fewer yH2AX foci were detected in dasatinib-pretreated LN fibroblasts 20 hours after irradiation, and after 40 hours, DNA damage was completely restored. These findings indicate that dasatinib pretreatment can increase repair of irradiation-induced DNA damage.

## Discussion

Previous studies have shown premature cellular aging in RA. Changes detected in RA patients include accelerated immunosenescence, decreased thymic functionality, telomeric attrition, and excessive senescence-associated secretory phenotype-related cytokine production [5, 9, 39]. In addition to many T-cell-related studies [8, 40], it was recently shown that senescent synovial fibroblasts prematurely accumulate in synovial tissue of RA patients [10]. Currently, it is unclear whether this premature aging is a primary event occurring before the onset of disease or whether it is a secondary event driven by inflammatory processes or treatment. Moreover, it is unknown whether premature aging originates in lymphoid organs crucial for immune cell differentiation and development and whether it can be observed in tissue-resident LN fibroblasts, which have a major influence on immune cell function.

In this study, we aimed to delineate the potential senescence phenotype of LN fibroblasts during the earliest phases of autoimmunity. To investigate the hallmarks of senescence during systemic autoimmunity in the absence of chronic inflammation, we compared LN fibroblasts collected from autoantibody-positive individuals at risk of developing RA with LN fibroblasts from RA patients and seronegative healthy volunteers. We detected senescence hallmarks in LN fibroblasts from RA patients at morphological, transcriptional, and functional levels compared with LN fibroblasts from age-matched controls. LN fibroblasts from RA-risk individuals also presented several hallmarks of senescence, although to a lesser extent, suggesting that senescent cell accumulation starts before the onset of clinical disease. The apparent absence of signs of chronic inflammation in synovial [11, 41] and LN [42] biopsies from RA-risk individuals suggests that the observed senescence phenotype is not related to inflammation. Whether the senescence is linked to future RA development remains to be investigated in follow-up studies.

Morphological characteristics related to senescence, such as enlarged and more granular cells, were significantly more common in RA LN fibroblasts *ex vivo*, and this morphology was maintained in culture, allowing functional *in vitro* studies. Moreover, cell size and granularity of cultured RA-risk LN fibroblasts were also greater than those of control LN fibroblasts, although to a lesser extent. The forward–sideward scatter plots suggest that a smaller number of larger (senescent) cells may be present in RA-risk individuals compared with RA LN fibroblasts, suggesting that accumulation of larger cells starts before the onset of RA and continues after diagnosis. Increased endogenous autofluorescence in senescent and slow-dividing cells has been attributed to lipofuscin accumulation in lysosomes as a result of oxidative stress and ROS production [32, 43, 44]. These lipofuscin granules contain highly oxidized proteins, mostly unsaturated fatty acids, that cannot be removed by proteasomal degradation and accumulate in lysosomes with age [29, 45]. We previously reported increased ROS production by RA LN fibroblasts [46], and here, we observed significantly more lipofuscin granules in RA-risk and RA LN fibroblasts. Taken together, these findings indicate that the autofluorescence detected in RA(-risk) LN fibroblasts may reflect increased lysosomal content and oxidative stress.

In senescent cells, increased lysosomal mass is partially reflected by increased SA-β-gal activity [45, 47]. SA-β-gal activity has been acknowledged as one of the first hallmarks of senescence, but has been extensively debated. Despite the high percentage of positivity in cultured RA LN fibroblasts, this finding is not necessarily representative of *in vivo* LN tissues. Most studies comparing *in vivo* and *ex vivo* SA-β-gal staining revealed a much lower percentage of positive cells in tissue, indicating that the culture process may influence SA-β-gal activity. However, our findings may still be relevant, as our age-matched control LN fibroblasts almost did not exhibit SA-β-gal-positive cells. Moreover, SenTraGor immunofluorescence staining was significantly greater in RA-risk LN fibroblasts than in control LN fibroblasts, pointing toward the accumulation of lysosomal content.

Transcriptomic analysis of senescence-associated genes in cultured LN fibroblasts revealed multiple genes that were differentially expressed between RA LN fibroblasts and controls. Overexpression of *FOXO4* mRNA was detected in RA LN fibroblasts and is suggested to prevent cell death of senescent cells by sequestering p53 in the nucleus. Notably, increased *NOTCH3* expression has been detected in RA-risk LN fibroblasts. Studies in inflamed synovium have shown that Notch3 is involved in the differentiation and arthrogenic expansion of a specific CD90+ fibroblast subset [48]. Another gene upregulated in RA LN fibroblasts is *CD38*. CD38 levels inversely correlate with the age-related decline in nicotinamide adenine dinucleotide (NAD), which ultimately leads to mitochondrial dysfunction and metabolic alterations [49, 50]. It is interesting to postulate that our earlier detected metabolic alteration in LN fibroblasts [46] is related to altered CD38 and NAD+ levels. Additional research is needed to investigate whether the differential expression of these genes is causatively related to the observed senescence phenotype of RA LN fibroblasts and may guide future studies aimed at restoring LN fibroblast function.

Cellular senescence has been described as a state of cell proliferation arrest. Although LN fibroblasts proliferate slowly, we did not detect proliferation arrest or significant differences between donor groups. However, the expression level of cyclin-dependent kinase inhibitor *CDKN2A* was significantly greater in RA LN fibroblasts compared with control LN fibroblasts. CDKN2A is one of the main drivers of cell cycle arrest in senescent cells [31]. The increased cell size measured using flow cytometry might explain why we were not able to detect any differences in cell proliferation, as this was measured using the IncuCyte, which uses confluence as a proliferation readout. When cells are larger, confluence is reached earlier compared with smaller cells; thus cell size is a confounding factor in our comparison of proliferation between control and RA LN fibroblasts. The scratch wound assay results showed that RA LN fibroblasts have a decreased migration capacity, as reflected by impaired wound repair, although this effect might also be caused by a difference in proliferation. Further research with larger sample numbers is needed to formally establish a difference in the proliferation rate between RA LN fibroblasts and controls. However, our data suggest that RA LN fibroblasts proliferate at a lower rate compared to healthy controls.

The accumulation of DNA damage or dysfunctional DNA repair machinery is linked to senescence and has been observed in T cells of patients with RA [40, 51–53]. T cells closely interact with resident fibroblasts within LN and synovial tissue, an interaction that might play an important role in initiating chronic inflammation. DNA damage is already present in LN fibroblasts of RA-risk individuals, thus occurring before the onset of disease. The persistence of DNA damage in senescent cells activates phosphorylation of transcription factor p53, which induces growth arrest to allow for DNA damage repair. Indeed, p53 expression was significantly upregulated in RA LN fibroblasts compared with controls. Additionally, DNA damage repair capacity was diminished in RA-risk and RA LN fibroblasts. Despite RA-risk and control LN fibroblasts having a similar number of yH2AX foci directly after irradiation, yH2AX foci persisted in RA-risk LN fibroblasts after irradiation. Further research should be undertaken to investigate whether impaired DNA damage repair correlates with future arthritis development in RA-risk individuals and what effect DNA damage in LN fibroblasts has on peripheral tolerance.

After four different senolytic agents were tested, only dasatinib was able to selectively target (senescent) LN fibroblasts from RA patients and did not affect controls. Dasatinib is primarily known for its ability to selectively eliminate senescent cells. Accordingly, following dasatinib treatment, we observe a reduction in cell density in the treated culture flasks. When evaluating cell viability upon dasatinib treatment, we showed that the viability of irradiated control LN fibroblasts increased after 48–72 hours, suggesting that irradiation-induced senescent cells were eliminated by dasatinib. The exponential curve after this time point suggests that healthy LN fibroblasts then had the opportunity to grow. This effect was less clear in dasatinib-treated RA LN fibroblasts. However, removal of senescent cells from the LN fibroblast pool by dasatinib treatment partly improves the overall cell size and gene expression levels in the pool of RA-risk LN fibroblasts. Dasatinib had only a minor effect on the lysosomal content of cultured LN fibroblasts. Data from baseline and irradiation experiments showed that dasatinib significantly improved irradiation-induced DNA damage but not the endogenous DNA damage observed in untreated RA(-risk) LN fibroblasts, suggesting that different repair mechanisms are involved. The mechanism by which dasatinib eliminates senescent cells from the LN fibroblast pool remains to be further elucidated. Dasatinib could have indirectly affected inflammatory signaling pathways. Senescent cells secrete pro-inflammatory cytokines, and because dasatinib reduces the number of senescent cells, this will have a positive impact on the remaining cells. As such, dasatinib may play a dual role in impacting the overall tissue fibroblast function by eliminating not only the senescent but also their pro-inflammatory environment. Thus, senolytic agents can be effective to improve the overall cell function of the LN fibroblast pool early in disease.

To our knowledge, this is the first study investigating cellular senescence in human LN fibroblasts during systemic autoimmunity. The greater number of senescent LN fibroblasts in RA patients than in controls suggests that, in addition to T cells and synovial fibroblasts, LN fibroblasts present signs of premature aging. Unfortunately, the relatively low number of donors tested in this study makes it difficult to reach statistical significance. However, these unique human LN biopsy studies are extremely challenging to perform, especially in the context of systemic autoimmunity. Moreover, it is difficult to find seronegative healthy volunteers who would like to participate in such a study, and LN fibroblasts are typically slowly growing cells, making these experiments very labor-intensive and time-consuming. A limitation of our study is that one type of senescence, known as replicative senescence, can easily be induced in *in vitro* culture systems. Replicative senescence is characterized by a decrease in the proliferation potential of cells after they undergo multiple rounds of cell division, leading to replicative exhaustion. However, we cautiously monitored our proliferation and passaging process and compared LN fibroblasts from different donors from the same passage after *ex vivo* digestion. Importantly, our LN biopsy data confirmed the increased cell size of RA fibroblasts directly analyzed *ex vivo* before *in vitro* expansion. Accordingly, these findings indicate that the observed senescence phenotype in RA(-risk) LN fibroblasts is not purely induced by *in vitro* culture. In addition, we excluded age as a confounding factor, as both the *ex vivo* and *in vitro* cell culture cohorts showed similar flow cytometry findings, despite the age differences among the analyzed RA patients. *In vitro* cultures mainly contain double negative and fibroblast reticular cells subtypes, distinguished by the expression of podoplanin (PDPN). Recently, it has been discussed that PDPN negative and PDPN positive LN fibroblasts can have similar characteristics and that the heterogeneous characteristics of LN fibroblasts are maintained in culture [54]. Although the senescent phenotype seems present in the majority of LN fibroblasts, further studies are needed to investigate their fibroblast phenotype.

## Conclusion

In conclusion, our results showed that RA LN fibroblasts exhibit many characteristics linked to cellular senescence. The removal of senescent cells by dasatinib treatment resulted in an overall improvement in the cell function of the RA(-risk) LN fibroblast pool. Although one of the most important hallmarks of senescence, proliferation arrest, was not detected, we postulate that LN fibroblasts from RA patients display a senescence phenotype that is partly present even before the onset of disease and contributes to the premature aging of the LN stromal microenvironment. Whether the observed LN fibroblast senescence is a causative factor in RA development or a secondary effect of immune dysregulation remains to be investigated. The presence of senescence in LN fibroblasts of RA-risk individuals suggests early involvement in autoimmunity, but longitudinal studies are needed to establish causality.

## Supplementary Material

uxaf029_suppl_Supplementary_Figure_S1

uxaf029_suppl_Supplementary_Figure_S2

uxaf029_suppl_Supplementary_Figure_S3

uxaf029_suppl_Supplementary_Figure_S4

uxaf029_suppl_Supplementary_Figure_S5

uxaf029_suppl_Supplementary_Figure_Legends

## Data Availability

All data are incorporated into the article and its online supplementary material.

## Abbreviations

ACPA: anti-citrullinated protein antibodies
LN: Lymph node
RA: rheumatoid arthritis
RA-risk: individuals who are positive for RF or/and ACPA and at risk of developing RA
RF: rheumatoid factor
