# Supplementary figures and images for: Senolytic treatment to rescue hallmarks of senescence in lymph node fibroblasts from patients with rheumatoid arthritis: Implications for premature aging and potential therapeutic intervention in early rheumatoid arthritis
Source: Clin Exp Immunol. 2025 May 8;219(1):uxaf029. doi: 10.1093/cei/uxaf029 (PMC12188290; doi:10.1093/cei/uxaf029)

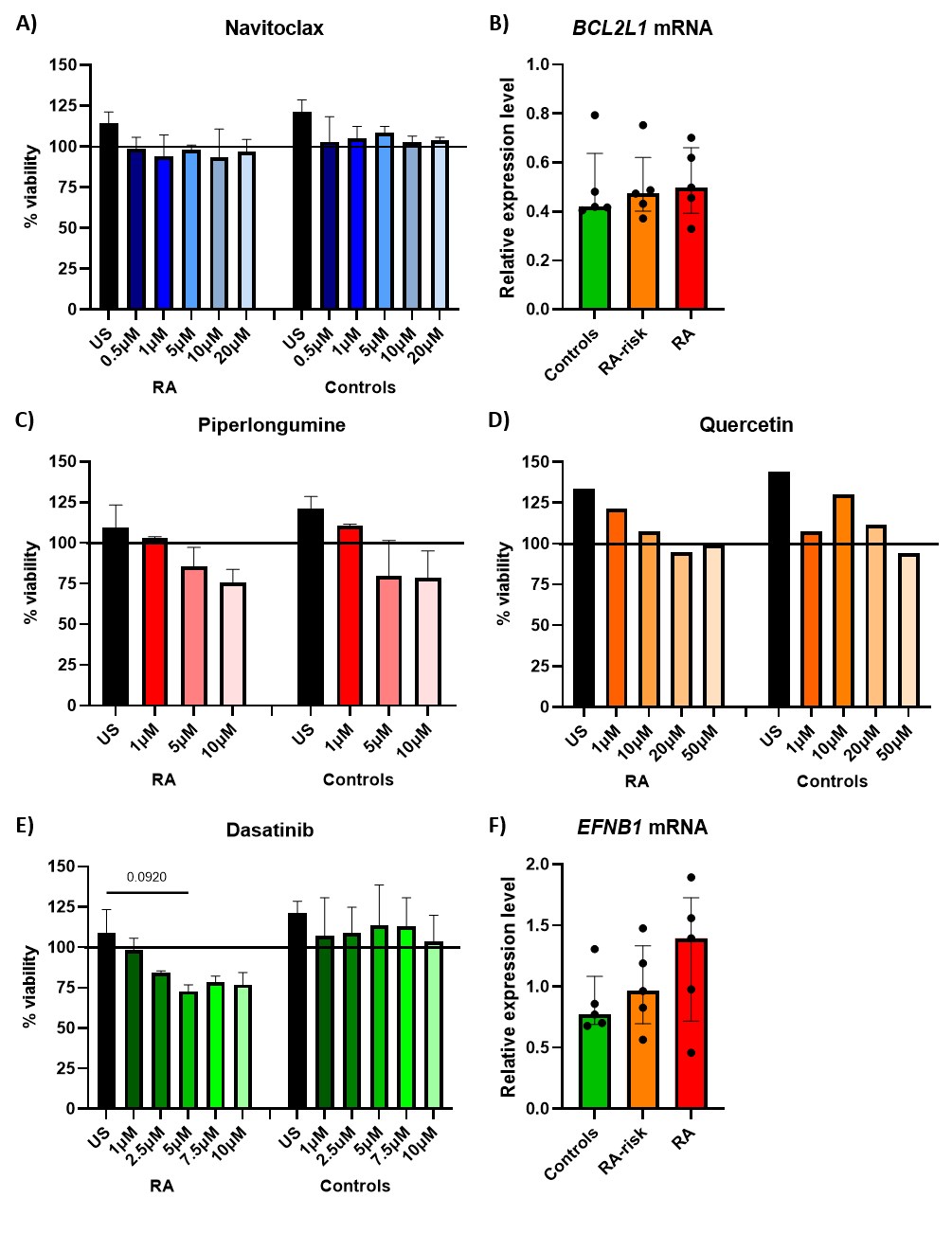

Supplement: uxaf029_suppl_Supplementary_Figure_S1 [file uxaf029_suppl_supplementary_figure_s1.jpeg]

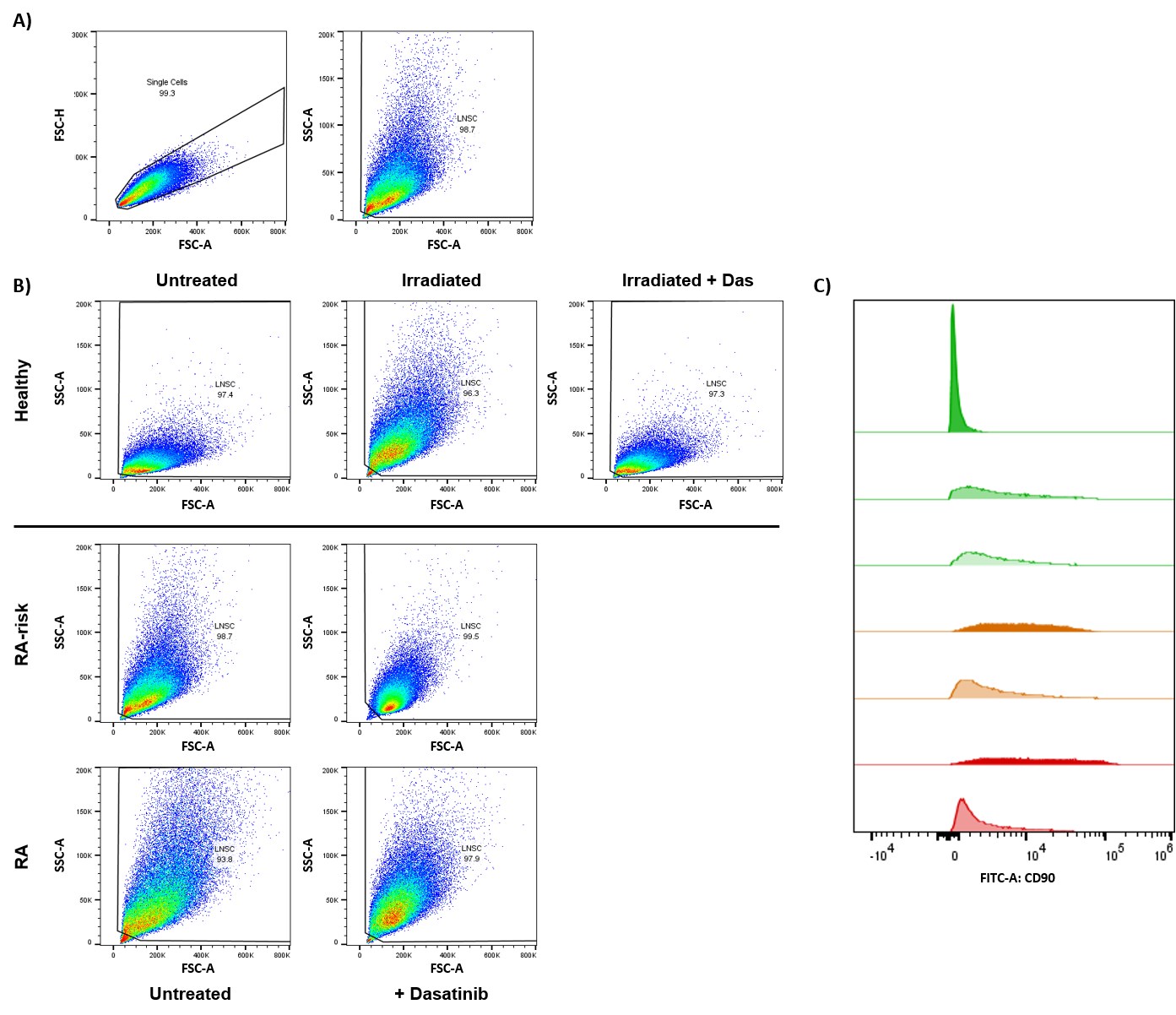

Supplement: uxaf029_suppl_Supplementary_Figure_S2 [file uxaf029_suppl_supplementary_figure_s2.jpeg]

## PARP1

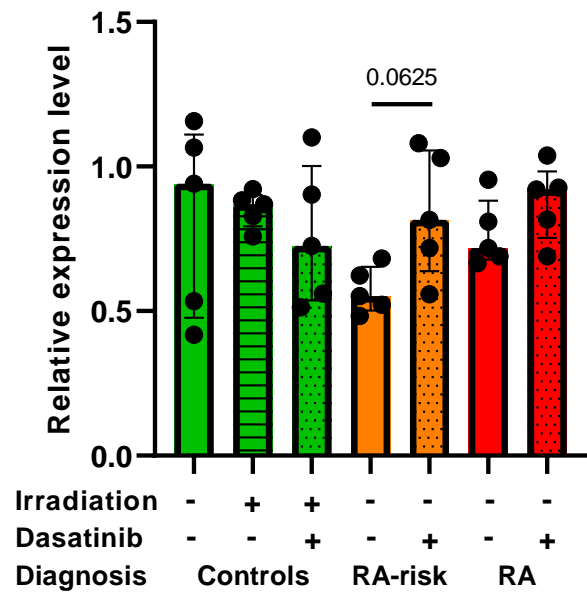

## NAMPT

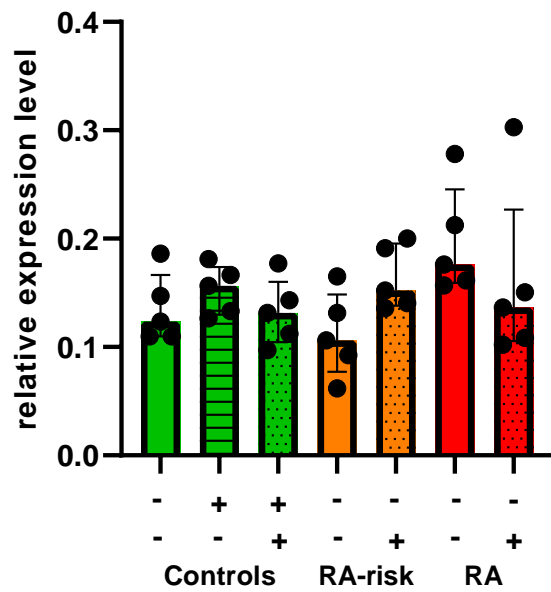

## SIRT1

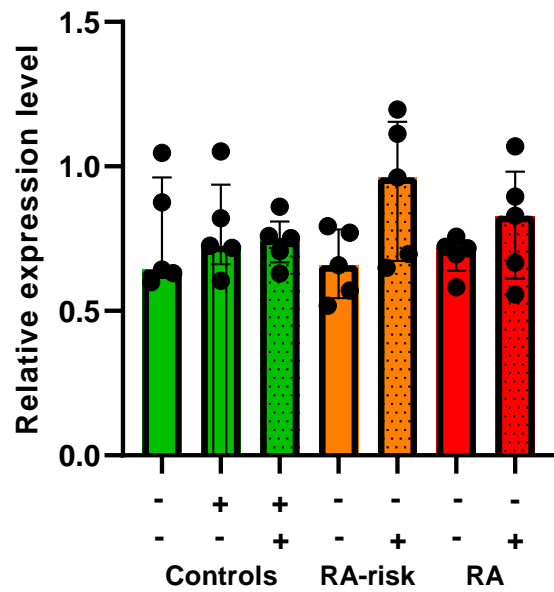

Supplement: uxaf029_suppl_Supplementary_Figure_S3 [file uxaf029_suppl_supplementary_figure_s3.pdf]

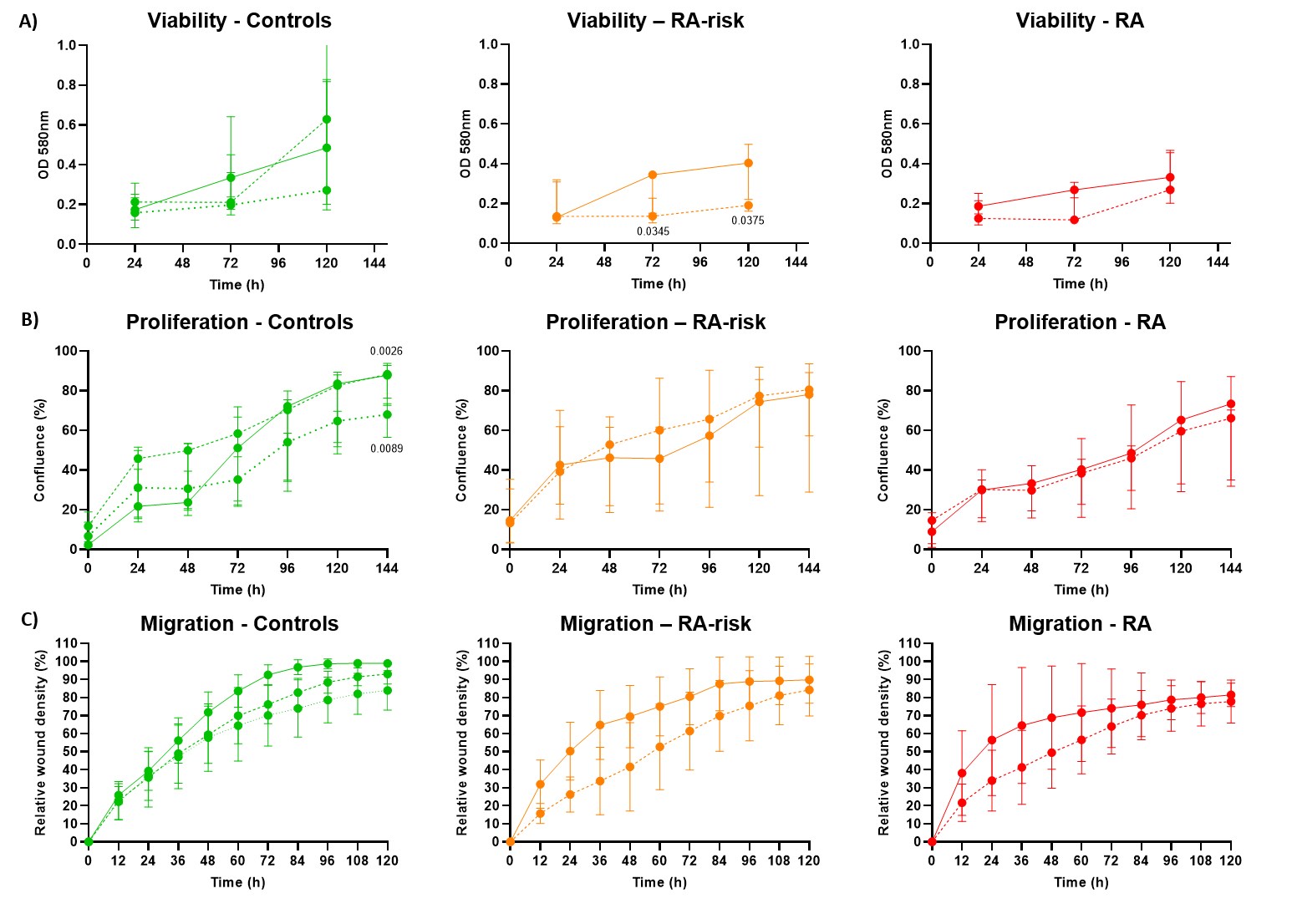

Supplement: uxaf029_suppl_Supplementary_Figure_S4 [file uxaf029_suppl_supplementary_figure_s4.jpeg]

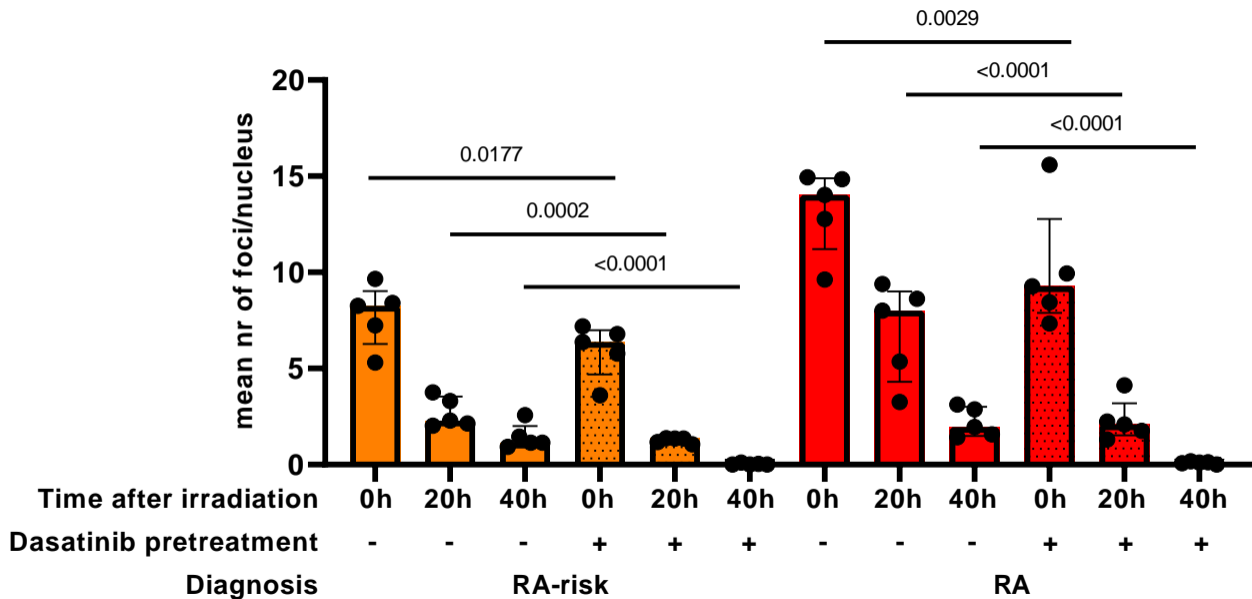

Supplement: uxaf029_suppl_Supplementary_Figure_S5 [file uxaf029_suppl_supplementary_figure_s5.pdf]
