# Supplementary material for: Senolytic treatment to rescue hallmarks of senescence in lymph node fibroblasts from patients with rheumatoid arthritis: Implications for premature aging and potential therapeutic intervention in early rheumatoid arthritis
Source: Clin Exp Immunol. 2025 May 8;219(1):uxaf029. doi: 10.1093/cei/uxaf029 (PMC12188290; doi:10.1093/cei/uxaf029)
Supplement: uxaf029_suppl_Supplementary_Figure_Legends [file uxaf029_suppl_supplementary_figure_legends.docx]

**Supplementary Figure 1. Selecting potential senolytics for LN fibroblasts.** A) Viability of RA and control LN fibroblasts after navitoclax treatment for 24 hours. B) Relative expression level of *BCL2L1* mRNA. A gene related to the antiapoptotic pathway of BCL-XL and a target of navitoclax. C) Viability of LN fibroblasts after piperlongumine treatment for 24 h. D) Viability of LN fibroblasts after quercetin treatment for 24 h. E) Viability of LN fibroblasts after dasatinib treatment for 24 h. Viability was measured via MTT assays. F) Relative expression level of *EFNB1* mRNA, a gene encoding for ephrin B1, which is a type I membrane protein and ligand for dasatinib. All donors were at passage 6 for qPCR analysis; N=5 per group. Data are presented as median + interquartile range. Statistical differences were determined using a Kruskal-Wallis followed by Dunn’s multiple comparisons test.

**Supplementary Figure 2. Gating strategy and flow cytometry plots of representative donors.** A) Flow cytometry gating strategy used to identify single cells and LN fibroblasts. Numbers adjacent to the outlined areas indicate percentages of cells in the gated population. B) Representative flow cytometry plots of irradiated and dasatinib (das) treated LN fibroblasts. C) Representative mean fluorescent intensity (MFI) plots of FITC autofluorescence in unstained LN fibroblasts. Controls (green), RA-risk individuals (orange) and RA patients (red).

**Supplementary Figure 3. Gene expression profiles in LN fibroblasts.** Gene expression levels of senescence-associated genes in cultured LN fibroblasts. All donors were at passage 6, N=5 per group. Data are presented as median + interquartile range. Statistical differences at baseline were determined using a Kruskal-Wallis test followed by Dunn’s multiple comparisons test, and a Wilcoxon matched pairs signed rank test was used to analyze the effect of dasatinib treatment.

**Supplementary Figure** **4. Dasatinib treatment significantly reduces cell viability of RA-risk LN fibroblasts.** A) Viability of irradiated and dasatinib-treated LN fibroblasts measured via MTT. B) Real-time cellular proliferation of irradiated and dasatinib-treated LN fibroblasts measured via IncuCyte software. C) Real-time cellular migration capacity of irradiated and dasatinib-treated LN fibroblasts measured via scratch wound assay software from IncuCyte. All donors passage 6, N=5 per group), with 3 technical replicates per condition for viability and 5 technical replicates for proliferation and migration assays. Data are presented as median + interquartile range. Statistical differences were determined using a repeated-measures ANOVA with the Geisser–Greenhouse correction followed by Dunnett’s multiple comparisons test.

**Supplementary Figure 5. Dasatinib treatment significantly improved irradiation-induced DNA damage repair in cultured LN fibroblasts.** A) Representative images of yH2AX foci (red) and DAPI staining (blue) in cultured LN fibroblasts. B) Representative images of yH2AX foci (red) and DAPI staining (blue) in cultured LN fibroblasts after DNA damage induction using gamma-irradiation. C) Average number of yH2AX foci per nucleus in cultured LN fibroblasts directly and 20 hours and 40 hours after irradiation. Mean value per donor was determined through quantification of Z-stack images of approximately 50 cells per donor. All donors were at passage 7, N=5 per group. Data are presented as median + interquartile range. Statistical differences were determined using 2-way ANOVA + Dunnett’s T3 multiple comparisons test.
